# Altered effector function of peripheral cytotoxic cells in COPD

**DOI:** 10.1186/1465-9921-10-53

**Published:** 2009-06-22

**Authors:** Richard A Urbanowicz, Jonathan R Lamb, Ian Todd, Jonathan M Corne, Lucy C Fairclough

**Affiliations:** 1 COPD Research Group, Institute of Infection, Immunity and Inflammation, The University of Nottingham, NG7 2UH, UK; 2 Immunology and Infection Section, Division of Cell and Molecular Biology, Faculty of Natural Sciences, Imperial College London, South Kensington, London, SW7 9AZ, UK; 3 Department of Respiratory Medicine, Nottingham University Hospitals, NG7 2UH, UK

## Abstract

**Background:**

There is mounting evidence that perforin and granzymes are important mediators in the lung destruction seen in COPD. We investigated the characteristics of the three main perforin and granzyme containing peripheral cells, namely CD8^+ ^T lymphocytes, natural killer (NK; CD56^+^CD3^-^) cells and NKT-like (CD56^+^CD3^+^) cells.

**Methods:**

Peripheral blood mononuclear cells (PBMCs) were isolated and cell numbers and intracellular granzyme B and perforin were analysed by flow cytometry. Immunomagnetically selected CD8+ T lymphocytes, NK (CD56^+^CD3^-^) and NKT-like (CD56^+^CD3^+^) cells were used in an LDH release assay to determine cytotoxicity and cytotoxic mechanisms were investigated by blocking perforin and granzyme B with relevant antibodies.

**Results:**

The proportion of peripheral blood NKT-like (CD56^+^CD3^+^) cells in smokers with COPD (COPD subjects) was significantly lower (0.6%) than in healthy smokers (smokers) (2.8%, p < 0.001) and non-smoking healthy participants (HNS) (3.3%, p < 0.001). NK (CD56^+^CD3^-^) cells from COPD subjects were significantly less cytotoxic than in smokers (16.8% vs 51.9% specific lysis, p < 0.001) as were NKT-like (CD56^+^CD3^+^) cells (16.7% vs 52.4% specific lysis, p < 0.001). Both cell types had lower proportions expressing both perforin and granzyme B. Blocking the action of perforin and granzyme B reduced the cytotoxic activity of NK (CD56^+^CD3^-^) and NKT-like (CD56^+^CD3^+^) cells from smokers and HNS.

**Conclusion:**

In this study, we show that the relative numbers of peripheral blood NK (CD56^+^CD3^-^) and NKT-like (CD56^+^CD3^+^) cells in COPD subjects are reduced and that their cytotoxic effector function is defective.

## Background

Chronic obstructive pulmonary disease (COPD) is a disease state characterised by progressive airflow limitation that is not fully reversible [1]. It is associated with an abnormal inflammatory response of the lungs to noxious particles or gases, primarily caused by cigarette smoking [2]. It is predicted to be the third most frequent cause of death worldwide by 2020 [3]. Although COPD is primarily a disease of the lungs there is now an appreciation that many of the manifestations of disease are outside the lung, such as cachexia, skeletal muscle dysfunction, depression and osteoporosis [4], leading to the concept that COPD is a systemic disease [5-9].

Many previous studies have examined functions of lung derived CD8+ T cells in patients with COPD, for example studies have shown an increase in CD8^+ ^cells within both the peripheral airway [10] and lower respiratory tract of the lungs of COPD patients [11-14]. It is known that lymphocytes can readily traffic between inflammatory sites (including lungs), regional lymph nodes, and importantly the systemic circulation, where they can be easily sampled [15] and hence may provide information using minimally invasive routes. This could be a benefit for subsequent biological and clinical investigations. To date research has been less conclusive in peripheral blood with some reporting an increase [12], a decrease in CD8+ cells [16] and others no change [17]. These conflicting findings may be due to the limits of some of the techniques employed and it is conceivable that other cell subpopulations expressing CD8 were included, i.e., CD8^+ ^natural killer (NK) cells (CD3^-^CD8^+^CD56^+^CD16^+/-^) and natural killer T (NK-T) cells (CD3^+^CD8^+^CD56^+^). Furthermore, CD8^+ ^T cells can be divided into three subtypes, namely memory, naïve and the highly cytotoxic effector memory cells (T_EMRA_), the latter of which is determined by their high perforin content [18]. To date no analysis has looked at the proportions of these subtypes.

The numbers of peripheral blood NK (CD56^+^CD3^-^) cells have been shown to be reduced in smokers with COPD compared to healthy volunteers and have reduced phagocytic activity [19], with parallel changes in NK cells reported in asymptomatic smokers [20]. In contrast no difference in NK cell numbers or functional activity has been found in lung parenchyma of patients with COPD [21], although a decrease has been seen in the bronchoalveolar lavage (BAL) of patients with chronic bronchitis, compared to healthy volunteers [13].

To date, no in-depth study of NKT cells in patients with COPD has been performed, although an increased number of a particular subset of NKT cells (Vα24-Vβ11 invariant-NKT cells) have been reported in asthma [22,23]. This, however, is still controversial, as others have not found the same increase [24]. Due to the extremely low number of invariant NKT cells in the peripheral blood [25] our analysis was expanded to include both invariant NKT cells and the TCR diverse non-invariant NKT cells by using CD3 and CD56 as markers [23]. NKT-like (CD56^+^CD3^+^) cells share both receptor structure and function of NK cells and T cells [26]. They can express T cell markers like CD3, CD4 and CD8 and NK cell markers like CD56, CD161 and inhibitory NK cell receptors (KIRs).

CD8^+ ^T lymphocytes, NK (CD56^+^CD3^-^) cells and NKT-like (CD56^+^CD3^+^) cells are all members of the 'professional killer' family that use the perforin/granzyme granule exocytosis pathway to cause targeted cell death. There is evidence that perforin and granzymes could play an important role in the lung tissue destruction witnessed in COPD [27-30] and contribute to the pathogenesis of the disease. This destruction may arise from either direct cell-cell interactions or exogenously present granzyme. Previous studies have identified an increased number of granzyme B positive cells in the peripheral blood [31] and an increased number of perforin positive cells in induced sputum of COPD subjects [28]. However, no functional assays or staining for double positive perforin/granzyme cells has been conducted on peripheral blood-derived cells.

Perforin is a Ca^2+^-dependent pore-forming protein that multimerizes in the plasma membrane of cells to form 5–20 nm pores [32]. These pores formed by perforin result in the collapse of the membrane potential. Water, ions and proteases of the cathepsin superfamily, most notably granzyme B, then enter the target cell and initiate the apoptotic cascade.

In humans, five granzymes with differing substrate specificity have been identified [33]. Granzyme B has the strongest apoptotic activity of all the granzymes as a result of its caspase-like ability to cleave substrates at aspartic acid residues thereby activating pro-caspases directly and cleaving downstream caspase substrates [34].

In this study therefore we investigated, within peripheral blood, the number and cytotoxic function of the three main classes of human killer cells; namely CD8^+ ^T lymphocytes, NK (CD56^+^CD3^-^) cells and NKT-like (CD56^+^CD3^+^) cells.

## Methods

### Study population and procedures

The Nottingham Local Research Ethics Committee approved the study protocol and written informed consent was obtained from the 46 participants before entering the blinded study. Of these, the 11 participants diagnosed as having COPD (COPD subjects), according to the ATS guidelines, were current smokers and had an FEV_1 _below 80% of predicted with an FEV_1_/FVC ratio of <70% and reversibility to an inhaled beta-2 agonist of <10% or <200 mls absolute improvement. 17 healthy smokers (smokers) and 18 non-smoking healthy participants (HNS), with an FEV_1 _above 80% of predicted, were recruited and matched for age and for the smokers, smoking history, as closely as possible. Table 1 details the demographic and spirometric data of the subjects used for the cell numbers and intracellular protein staining. Table 2 details the demographic and spirometric data of the subjects that were used for the cytotoxicity assay, which included 4 HNS, 8 smokers and 9 COPD subjects from the previous part of the study. Participants were excluded if they had a history of physician diagnosed asthma or a positive skin prick test response to any of the following allergens: grass pollen, house dust mite, cat dander and dog hair (ALK-Abelló). COPD subjects were also excluded if they had had an exacerbation within the previous 6 weeks, were α1-anti-trypsin deficient or had lung cancer. Six out of ten COPD subjects had received inhaled corticosteroids within 6 weeks of entering the study.

**Table 1:** Demographic and spirometric values of the studied groups

	**HNS**	**Smokers**	**COPD subjects**
**Subjects**	12	15	10
**Age (years)**	52 (42–68)	60 (42–68)	66 (56–72)
**Gender (M/F)**	3/9	7/8	6/4
**Packs/yrs**	0 (0–0)	36 (15–95)	51 (24–72)
**FEV_1 _(% pred)**	106 (93–140)	95 (81–116)	46 (17–71)
**FEV_1_/FVC (%)**	79 (68–86)	76 (67–86)	47 (32–66)
**BMI (kg/m^2^)**	23.6 (18.9–32.0)	23.9 (19.9–36.9)	26.1 (19.3–35.6)
**MRC dyspnoea scale**	N/A	N/A	3 (2–4)
**Distance walked in 6 min (m)**	N/A	N/A	347 (141–494)
**BODE Index**	N/A	N/A	6 (2–9)

Results are expressed as median with range in brackets.

HNS, Healthy non-smokers; COPD, chronic obstructive pulmonary disease; FEV_1_, forced expiratory volume in 1 second; pred, predicted value; FVC, forced vital capacity.

**Table 2:** Demographic and spirometric values of the studied groups for the cytotoxicity assay

	**HNS**	**Smokers**	**COPD subjects**
**Subjects**	10	10	10
**Age (years)**	67 (45–75)	61 (43–67)	64 (56–72)
**Gender (M/F)**	3/7	3/7	5/5
**Packs/yrs**	0 (0)	36 (15–95)	43 (24–68)
**FEV_1 _(% pred)**	106 (93–132)	88 (81–115)	46 (17–68)
**FEV_1_/FVC (%)**	77 (71–85)	72 (67–86)	48 (31–66)
**BMI (kg/m^2^)**	25.3 (18.9–32.0)	24.3 (20.0–36.8)	26.1 (19.3–35.6)
**MRC dyspnoea scale**	N/A	N/A	4 (2–4)
**Distance walked in 6 min (m)**	N/A	N/A	265 (141–494)
**BODE Index**	N/A	N/A	6 (2–9)

Results are expressed as median with range in brackets.

HNS, Healthy non-smokers; COPD, chronic obstructive pulmonary disease; FEV_1_, forced expiratory volume in 1 second; pred, predicted value; FVC, forced vital capacity.

### PBMC isolation and fractionation

Peripheral blood mononuclear cells (PBMCs) were isolated from whole blood on a discontinuous Histopaque density gradient (Sigma). NK (CD56^+^CD3^-^) and NKT-like (CD56^+^CD3^+^) cells were isolated from PBMCs using a CD56 multi-sort kit in conjunction with α-CD3 microbeads (Miltenyi Biotech Ltd) according to manufacturers' instructions. Briefly, PBMCs were incubated for 15 minutes at 4°c with α-CD56 MultiSort microbeads and separated on a refrigerated MS column using cold PBS containing 1% FCS and 0.4% EDTA. The resulting positive fraction was then incubated with MultiSort Release Reagent for 10 minutes at 4°C, washed and then incubated with MultiSort Stop Reagent and α-CD3 microbeads for 15 minutes at 4°C. Finally the labelled cells were separated on a refrigerated LS column. CD8^+ ^T lymphocytes (CD8^+^CD56^-^) were positively selected from the CD56 negative fraction with α-CD8 microbeads. Following isolation, all fractions were washed, counted and purity confirmed at ≥ 94% by flow cytometric analysis.

### Flow cytometric analysis

Cells were washed with PBA, fixed in 3% formaldehyde in isotonic azide free solution and given a final wash with PBA – 0.04% saponin with 10% FCS. Labelled antibodies (Table 3) were added at the recommended concentration and the cells were incubated for two hours at 4°C in the dark. Excess antibody was removed by washing and cells were stored in 0.5% formaldehyde in isotonic azide free solution at 4°C. Flow cytometric analysis of these antibody labelled cells was performed using an EPICS Altra (Beckman Coulter). Fifty thousand live-gated events were collected for each sample and isotype matched antibodies were used to determine binding specificity. Data were analysed using WEASEL version 2.3 (WEHI). Dead cells were excluded from analysis according to their forward and side scatter characteristics.

**Table 3:** Antibodies used for flow cytometry and blocking experiments

**Antigen**	**Fluorochrome**	**Isotype**	**Clone**	**Company**
CD3	ECD PC7	Mouse IgG1	UCHT1	Beckman Coulter, Luton, UK
CD4	FITC PC5	Mouse IgG1	13B8.2	Beckman Coulter, Luton, UK
CD8	PC5 APC	Mouse IgG1	B9.11	Beckman Coulter, Luton, UK
CD8	ECD	Mouse IgG1	SFCl21Thy2D3	Beckman Coulter, Luton, UK
CD14	FITC	Mouse IgG2a	RM052	Beckman Coulter, Luton, UK
CD16	PC7	Mouse IgG1	3G8	Beckman Coulter, Luton, UK
CD19	PC5	Mouse IgG1, k	J4.119	Beckman Coulter, Luton, UK
CD45RA	FITC PE	Mouse IgG1	ALB11	Beckman Coulter, Luton, UK
CD45RO	ECD	Mouse IgG2a	UCHL1	Beckman Coulter, Luton, UK
CD56	PE PC5 PC7	Mouse IgG1	N901	Beckman Coulter, Luton, UK
CD62L	PC5	Mouse IgG1	DREG56	Beckman Coulter, Luton, UK
Granzyme B	FITC	Mouse IgG1k	GB11	Becton Dickinson, Oxford, UK
Perforin	PE	Mouse IgG2b	δG9	Becton Dickinson, Oxford, UK
Granzyme B	N/A	Mouse IgG2a	2C5/F5	Becton Dickinson, Oxford, UK
Perforin	N/A	Mouse IgG2b	δG9	Becton Dickinson, Oxford, UK

ECD, phycoerythrin-Texas Red-x; FITC, fluorescein isothiocyanate; PC5, phycoerythrin-cyanin 5.1; PC7, phycoerythrin-cyanin 7; PE, phycoerythrin

### Cytotoxicity assay

The cytotoxic activities of NK cells (CD56^+^CD3^-^), NKT-like cells (CD56^+^CD3^+^) and CD8^+ ^T lymphocytes (CD8^+^CD56^-^) were determined by colorimetric quantification of lactate dehydrogenase (LDH) released from lysed target cells. A commercially available kit (CytoTox 96 Non-Radioactive Cytotoxicity Assay, Promega) was used with erythroleukaemic K562 cells (ECACC) as the target cell line. Briefly, the effector and target cells were mixed at a ratio of 5:1, plated, in quadruplicate, on a 96-well U-bottomed plate and incubated for 4 h at 37°C in a humidified atmosphere containing 5% CO_2_. After incubation, the samples were centrifuged, the supernatants collected and incubated for 30 min at room temperature with the Substrate Mix provided with the kit to detect LDH activity. A stop solution was added and the absorbance of the sample was measured at 490 nm on an Emax precision microplate reader (Molecular Devices) using SOFTmax software (Molecular Devices). The amount of cell-mediated cytotoxicity was calculated by subtracting the spontaneous LDH released from the target and effector cells from the LDH released by lysed target cells, using the following equation:



For the blocking experiments, immunomagnetically selected CD56^+ ^cells were incubated for 30 minutes with different concentrations of antibodies (Table 3) and used at the effector:target ratio of 5:1 against K562 cells in the LDH release cytotoxicity assay, as previously detailed.

### Statistical analysis

The statistical analysis was performed with Prism software, version 4.0c (GraphPad). Normality was detected using the Kolmogorov-Smirnov test. As some data were non-normally distributed all are expressed as median (range), unless otherwise stated. Differences between the three groups of subjects were tested using the non-parametric Kruskal-Wallis test with *post hoc *pairwise comparisons made by the Dunn's Multiple Comparison test to determine which pair was statistically significantly different. P values of less than 0.05 were considered to indicate statistical significance.

## Results

### Cellular constituents of peripheral blood

**Figure 1:**
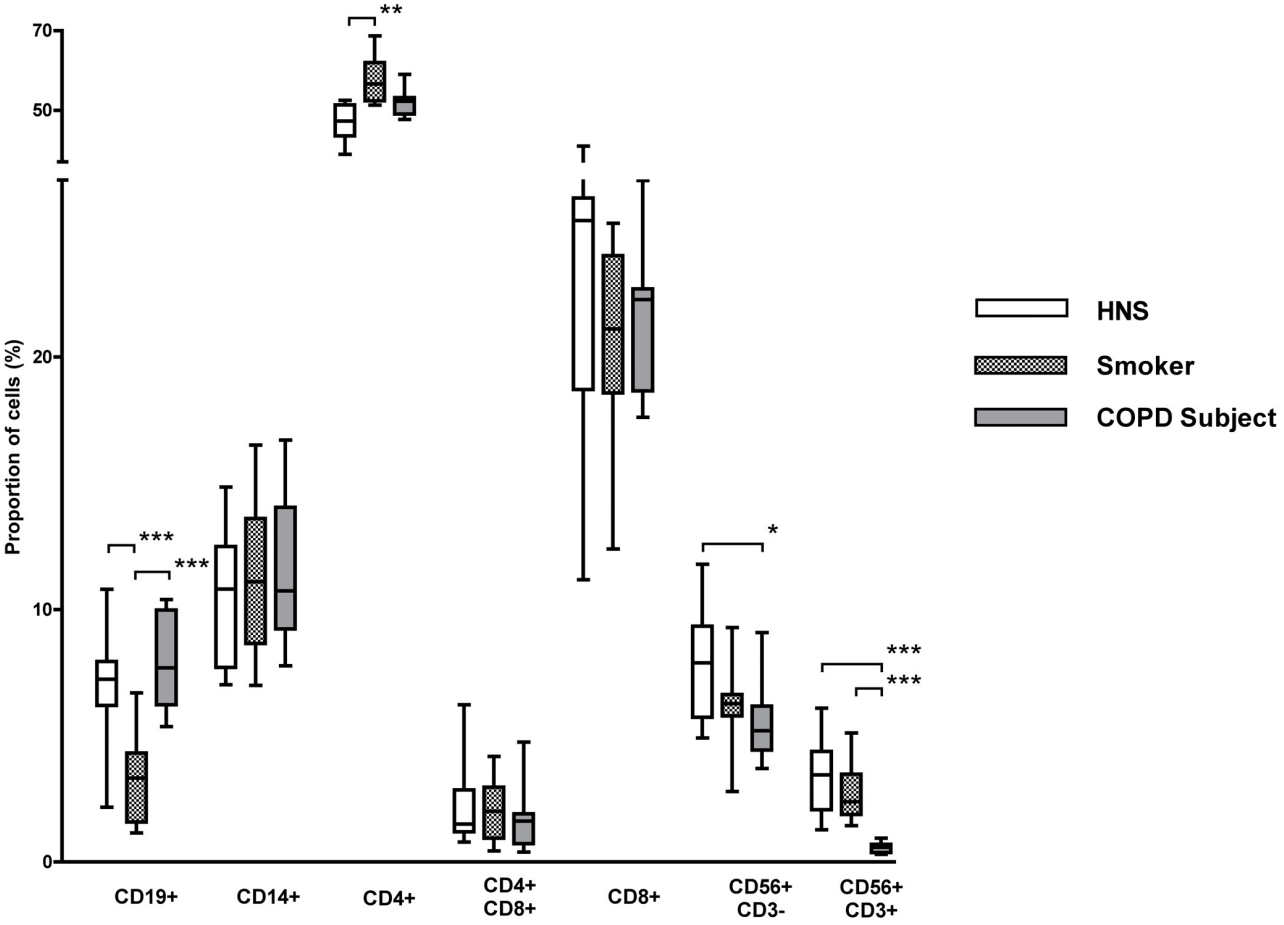
**Proportion and type of peripheral blood mononuclear cells in HNS (n = 12), smokers (n = 15) and COPD subjects (n = 10)**. Results show a significant decrease in the proportion of NK (CD56^+^CD3^-^) cells (*; p < 0.05) and NKT-like (CD56^+^CD3^+^) cells (***; p < 0.001) in COPD subjects compared to HNS. Smokers had a significantly lower proportion of B-cells (***; p < 0.001) compared to the other two groups and a greater proportion of CD4^+ ^T helper cells. Cell types were determined by flow cytometric analysis of monoclonal antibodies. CD19, B cells; CD4, T helper cells; CD8, cytotoxic killer cells; CD56^+^CD3^-^, NK cells; CD56^+^CD3^+^, NKT-like cells.

All individuals had similar total mononuclear cell numbers, which were within the normal lymphocyte range; therefore relative proportions of cell types were used for comparisons (Figure 1). The proportion of NKT-like (CD56^+^CD3^+^) cells in COPD subjects (0.6%) was significantly lower than in both smokers (2.8%; p < 0.001) and HNS (3.3%; p < 0.001).

**Figure 2:**
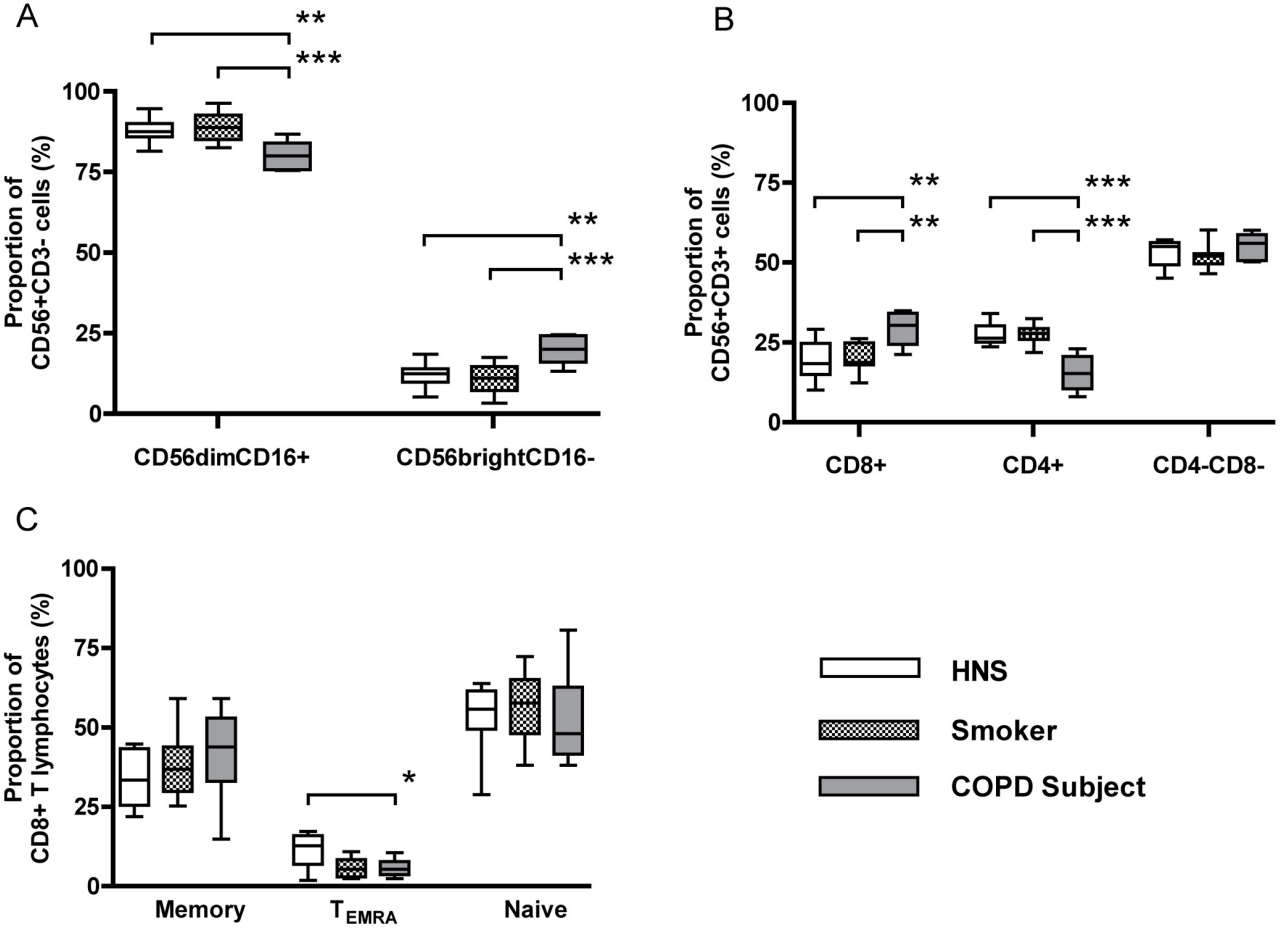
**Proportion of NK (CD56^+ ^CD3^-^) subsets (Panel A), NKT-like (CD56^+ ^CD3^+^) subsets (Panel B) and CD8+ T lymphocyte subsets (Panel C), from the peripheral blood of HNS (n = 12), smokers (n = 15) and COPD subjects (n = 10)**. Panel A shows the proportion of CD56^bright^CD16^- ^NK cells was significantly increased in COPD subjects compared to HNS (**; p < 0.01) and smokers (***; p < 0.001). Panel B shows significantly more CD8^+^CD56^+^CD3^+ ^cells (**; p < 0.01) in the peripheral blood of COPD subjects compared to the other two groups and a significant decrease in the proportion of CD4^+^CD56^+^CD3^+ ^cells (***; p < 0.001). Panel C shows cells of the highly cytotoxic effector memory phenotype (T_EMRA_; CD8^+^CD45RO^+^RA^+^CD62L^-^) were significantly decreased in COPD subjects (*; p < 0.05).

The proportion of NK (CD56^+^CD3^-^) cells was significantly lower in COPD subjects (5.5%) compared to HNS (7.9%; p < 0.01) (Figure 1). As well as a reduction in the overall proportion of NK (CD56^+^CD3^-^) cells in the peripheral blood of COPD subjects, the proportion of NK cells in the cytotoxic CD56^dim^CD16^+ ^subset was decreased (79.9%) compared to smokers (88.7%; p < 0.001) and HNS (88.6%; p < 0.01; Figure 2A), with a corresponding rise in the proportion of immunoregulatory CD56^bright^CD16^- ^cells in COPD subjects. No significant differences were observed in the proportion of CD8^+ ^CD56^dim^CD16^+ ^cells in COPD subjects or in the CD8^+ ^CD56^bright^CD16^- ^cells (data not shown).

Analysis of the NKT-like (CD56^+^CD3^+^) subsets revealed differences between the three groups (Figure 2B). In COPD subjects, the proportion of CD8^+ ^CD56^+^CD3^+ ^cells was significantly increased (29.2%) in relation to smokers (21.5%; p < 0.01) and HNS (19.7%; p < 0.01). There was a significant decrease in the number of CD4^+ ^CD56^+^CD3^+ ^cells in COPD subjects (15.7%) compared to smokers (27.4%; p < 0.001) and HNS (27.9%; p < 0.001) but no significant difference in the double negative (DN, CD3^+^CD4^-^CD8^-^CD56^+^) subset was detected.

As previously shown, there was no significant difference in the proportion of CD8^+ ^T lymphocytes between the three groups (Figure 1). However, further analysis of the CD8^+ ^T lymphocytes by flow cytometry revealed that the proportion of CD45RO^+^RA^+ ^(T_EMRA _cells) was significantly lower in both smokers and COPD subjects, compared to HNS (p < 0.05). COPD subjects had a trend of more memory cells (CD8^+^CD45RO^+^RA^-^) and a corresponding reduction in the proportion of naïve cells (CD8^+^CD45RO^-^RA^+^) compared to the other two groups (Figure 2C), although this did not reach significance.

### Expression of cytotoxic effector molecules

**Figure 3:**
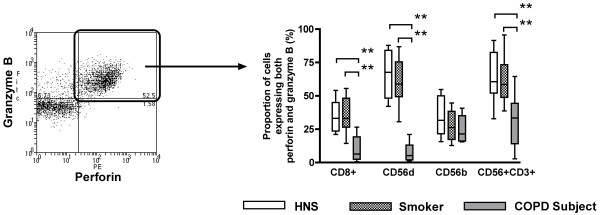
**Representative flow cytometry plot (Panel A) showing the expression of both granzyme B and perforin (Panel B) in CD88^+ ^T lymphocytes, CD56^dim ^CD16^+ ^NK cells, CD56^bright ^CD16^- ^NK cells and NKT-like (CD56^+^CD3^+^) cells from non-smoking healthy participants (n = 12), healthy smokers (n = 15) and smokers with COPD (n = 10)**. Double stained cells (Panel B) are deemed cytotoxic (**; p < 0.01).

The expression of perforin and granzyme B were studied in CD8^+ ^T lymphocytes, CD56^dim^CD16^+ ^NK cells, CD56^bright^CD16^- ^NK cells and NKT-like (CD56^+^CD3^+^) cells (Figure 3).

The proportions of CD8^+ ^T lymphocytes, CD56^dim^CD16^+ ^NK cells and NKT-like (CD56^+^CD3^+^) cells that expressed both perforin and granzyme B were significantly lower in COPD subjects (6.4%, 5.2% and 33.4%, respectively) than in smokers (33.0%; p < 0.01, 58.9%; p < 0.01 and 58.6%; p < 0.01) and HNS (33.2%; p < 0.01, 67.7%; p < 0.01 and 60.7%; p < 0.01) (Figure 3B). There was no difference in the proportion of CD56^bright^CD16^- ^NK cells expressing both granzyme B and perforin (Figure 3B).

The proportion of CD8^+ ^T lymphocytes that expressed only granzyme B and no perforin were significantly lower in COPD subjects (5.1%) compared to smokers (12.8%; p < 0.01) and HNS (12.7%; p < 0.01). No significant difference was observed between the proportions of CD56^dim^CD16^+ ^NK cells or CD56^bright^CD16^- ^NK cells expressing only granzyme B and no perforin between the three groups (data not shown). The proportion of NKT-like (CD56^+^CD3^+^) cells from COPD subjects that express only granzyme B and no perforin were significantly higher (10.7%) than smokers (3.4%; p < 0.01) and HNS (4.7%; p < 0.01).

The proportion of CD8^+ ^T lymphocytes that expressed only perforin and no granzyme B were significantly lower in COPD subjects (1.8%) compared to smokers (10.9%; p < 0.01) and HNS (7.9%; p < 0.01). There were significantly more CD56^dim^CD16^+ ^NK cells and NKT-like (CD56^+^CD3^+^) cells expressing only perforin and no granzyme B in COPD subjects (63.5% and 28.4%, respectively) compared to smokers (27.3%; p < 0.01 and 10.1%; p < 0.01) and HNS (32.2%; p < 0.01 and 7.0%; p < 0.01). No differences were observed in the proportion of CD56^bright^CD16^- ^NK cells expressing only perforin and no granzyme B between the three groups (data not shown).

### Cytotoxic activity of NK (CD56^+^CD3^-^), NKT-like (CD56^+^CD3^+^) cells and CD8^+ ^T lymphocytes

To establish if the different levels of expression of perforin and granzyme B in these cell populations would reflect their cytotoxic activity, NK (CD56^+^CD3^-^) cells, NKT-like (CD56^+^CD3^+^) cells and CD8^+ ^T lymphocytes were immunomagnetically purified from peripheral blood and screened in an LDH release assay. All samples were ≥ 94% pure with respect to B-lymphocytes, helper T lymphocytes, monocytes, neutrophils and each other (Tables 4, 5 and 6).

**Table 4:** Purity of immunomagnetically separated NK (CD56^+^CD3^-^) cells from the peripheral blood of the studied groups.

	**HNS**	**Smokers**	**COPD subjects**
**NK (CD56^+^CD3^-^) cells**	**96.2 (± 1.4)**	**96.6 (± 0.8)**	**96.8 (± 0.6)**
**NKT-like (CD56^+^CD3^+^) cells**	1.2 (± 0.6)	1.0 (± 0.9)	0.8 (± 1.2)
**Cytotoxic T cells (CD8^+^)**	0.4 (± 1.3)	0.7 (± 0.5)	0.6 (± 0.7)
**B cells (CD19^+^)**	0.9 (± 0.9)	0.5 (± 0.8)	0.9 (± 0.9)
**Helper T cells (CD4^+^)**	1.1 (± 0.3)	0.9 (± 0.2)	0.4 (± 1.0)
**Monocytes (CD14^+^)**	0.2 (± 1.1)	0.3 (± 0.4)	0.5 (± 1.3)

Results are expressed as mean with standard deviation in brackets.

**Table 5:** Purity of immunomagnetically separated NKT-like (CD56^+^CD3^+^) cells from the peripheral blood of the studied groups.

	**HNS**	**Smokers**	**COPD subjects**
**NK (CD56^+^CD3^-^) cells**	1.9 (± 1.2)	1.4 (± 0.3)	1.7 (± 1.3)
**NKT-like (CD56^+^CD3^+^) cells**	**95.6 (± 0.8)**	**96.3 (± 0.5)**	**96.6 (± 1.0)**
**Cytotoxic T cells (CD8^+^)**	0.5 (± 0.8)	0.2 (± 0.8)	0.6 (± 0.9)
**B cells (CD19^+^)**	0.7 (± 0.6)	1.1 (± 0.1)	0.4 (± 0.6)
**Helper T cells (CD4^+^)**	1.2 (± 1.3)	0.8 (± 0.3)	0.3 (± 1.2)
**Monocytes (CD14^+^)**	0.1 (± 1.1)	0.2 (± 0.9)	0.4 (± 1.0)

Results are expressed as mean with standard deviation in brackets.

**Table 6:** Purity of immunomagnetically separated CD8^+^T lymphocytes from the peripheral blood of the studied groups.

	**HNS**	**Smokers**	**COPD subjects**
**NK (CD56^+^CD3^-^) cells**	2.0 (± 0.5)	1.4 (± 1.4)	1.2 (± 1.0)
**NKT-like (CD56^+^CD3^+^) cells**	1.5 (± 1.1)	0.8 (± 1.2)	0.9 (± 1.2)
**Cytotoxic T cells (CD8^+^)**	**94.7 (± 1.4)**	**95.0 (± 1.2)**	**96.0 (± 0.4)**
**B cells (CD19^+^)**	0.2 (± 0.8)	0.9 (± 0.1)	0.8 (± 0.9)
**Helper T cells (CD4^+^)**	0.9 (± 0.9)	1.1 (± 1.0)	0.6 (± 0.6)
**Monocytes (CD14^+^)**	0.7 (± 0.8)	0.8 (± 0.7)	0.5 (± 1.0)

Results are expressed as mean with standard deviation in brackets.

**Figure 4:**
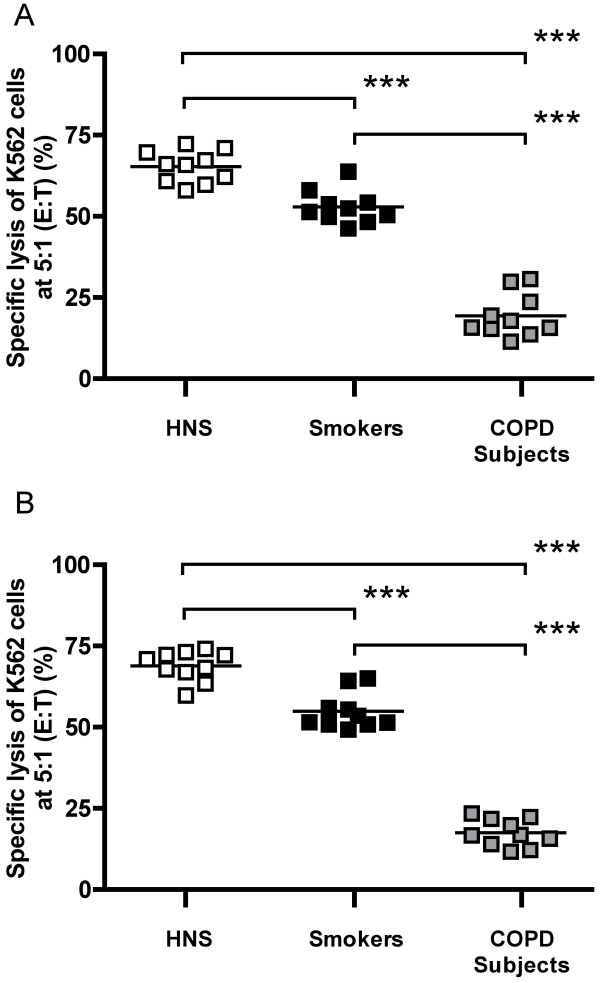
**Cytotoxic activity of NK (CD56^+ ^CD3^-^) cells (Panel A) and NKT-like (CD56^+ ^CD3^+^) cells (Panel B)**. Immunomagnetically separated cells (25,000) were cultured with K562 cells (5,000) giving an effector:target ratio of 5:1, in an LDH release cytotoxicity assay (***; p < 0.001).

Using the same number of effector cells (effector to target ratio of 5:1), both NK (CD56^+^CD3^-^) cells and NKT-like (CD56^+^CD3^+^) cells from COPD subjects were significantly less cytotoxic (16.8% and 16.7% specific lysis, respectively) than those from smokers (51.9%; p < 0.001 and 52.5%; p < 0.001) and HNS (66.0%; p < 0.001 and 69.6%; p < 0.001) (Figure 4A and 4B). NK (CD56^+^CD3^-^) cells and NKT-like (CD56^+^CD3^+^) cells from smokers were also significantly less cytotoxic than those from HNS (p < 0.001) (Figure 4A and 4B). As expected, due to K562 cells not expressing MHC class I, the CD8^+ ^T lymphocytes did not show any killing activity in this assay (data not shown).

**Figure 5:**
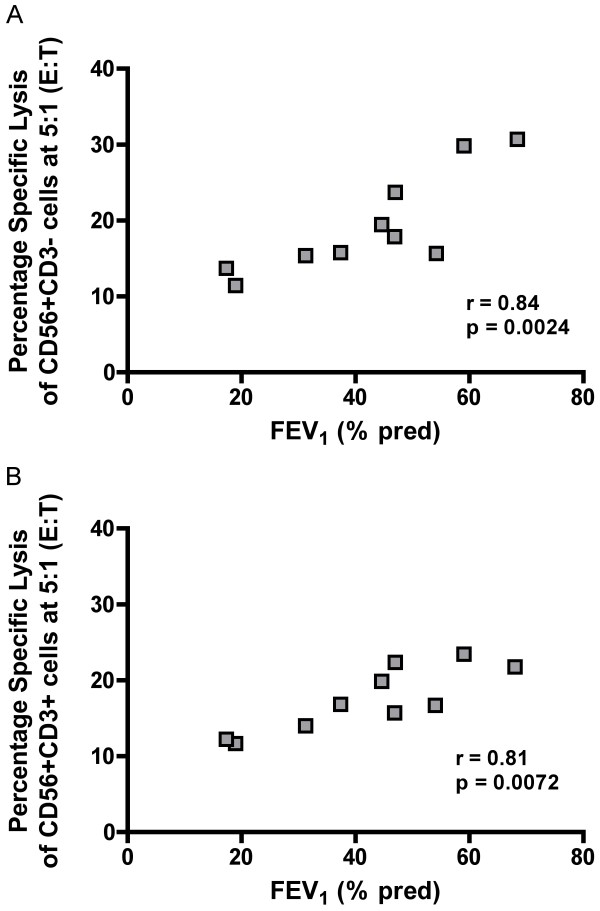
**Correlation of cytotoxic activity and lung function in NK (CD56^+^CD3^-^) cells (Panel A) and NKT-like (CD56^+^CD3^+^) cells (Panel B) in COPD subjects**. Immunomagnetically separated cells (25,000) were cultured with K562 cells (5,000) giving an effector:target ratio of 5:1, in an LDH release cytotoxicity assay compared to FEV_1 _(% pred).

The cytotoxic activity of both NK (CD56^+^CD3^-^) cells and NKT-like (CD56^+^CD3^+^) cells from COPD subjects correlated with lung function as assessed by FEV_1 _measurement (r = 0.84; p = 0.0024 and r = 0.81; p = 0.0072, respectively; Figure 5A and 5B).

### Blocking of cytotoxic activity

**Figure 6:**
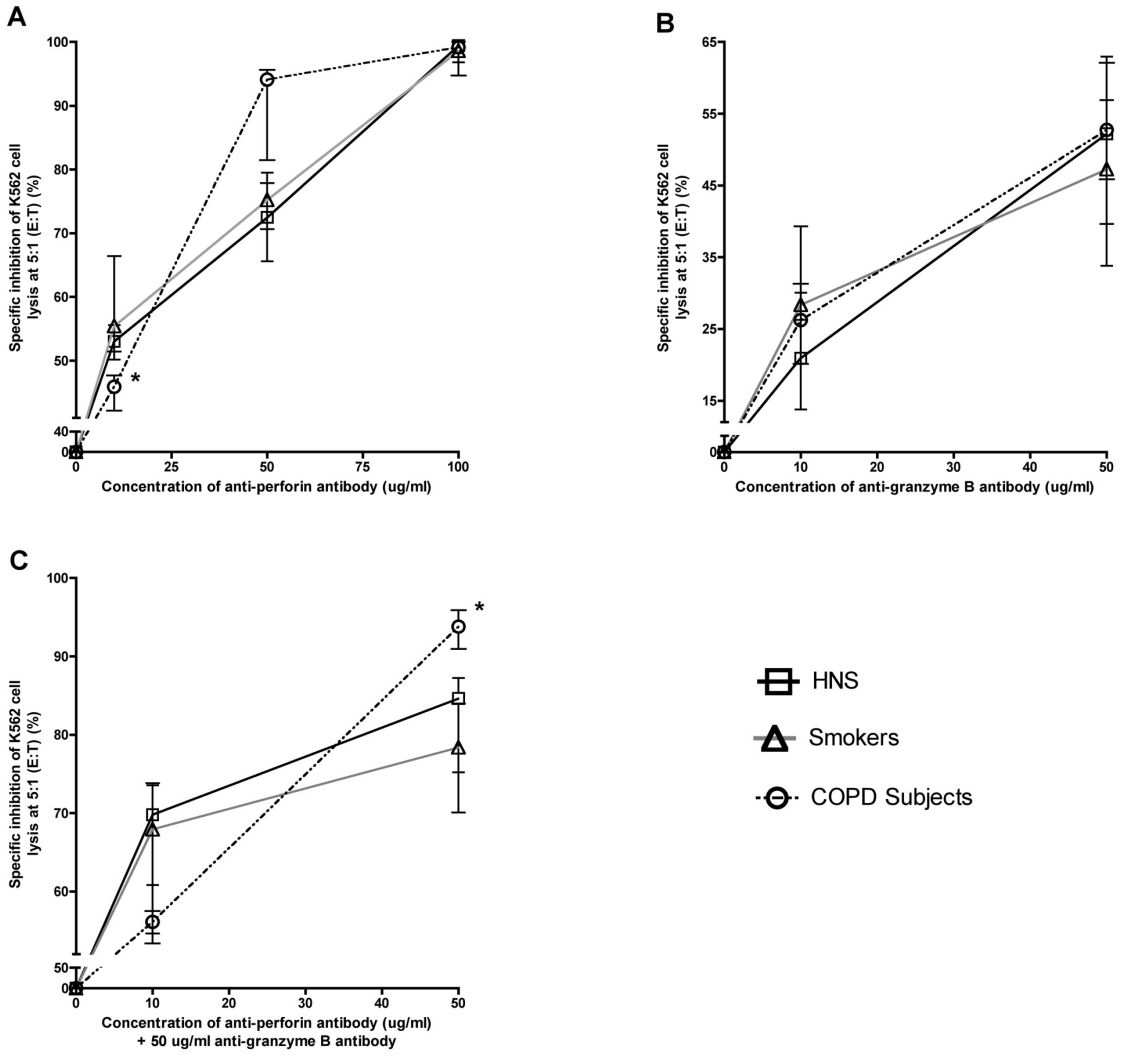
**Cytotoxic activity of CD56^+ ^cells in the presence of different concentrations of an anti-perforin antibody (A), an anti-granzyme B antibody (B) and a combination of the two (C) from HNS (n = 3), smokers (n = 3) and COPD subjects (n = 3)**. Immunomagnetically selected CD56^+ ^cells were incubated with the stated concentration of antibody and used at the effector:target ratio of 5:1 against K562 cells in the LDH release cytotoxicity assay.

In order to establish that the observed cytotoxicity of the NK (CD56^+^CD3^-^) and NKT-like (CD56^+^CD3^+^) cells was perforin and granzyme B dependent, the effector and target cells were incubated with differing concentrations of anti-perforin and anti-granzyme B antibodies alone and in combination. A dose-dependent inhibition of cytotoxic activity of CD56^+ ^cells was observed in all three groups. Total inhibition in COPD subjects occurred with a lower concentration of anti-perforin antibody (50 μg/ml) than in HNS and smokers (100 μg/ml; Figure 6A), although this was not statistically significant. The anti-granzyme B antibody had a limited inhibition effect on its own (Figure 6B), but increased the level of inhibition when combined, at 50 μg/ml, with perforin (Figure 6C).

Regression analysis confirmed that all the key differences reported here, between the COPD subjects, the smokers and HNS, remained, even after adjusting for age, gender and inhaled corticosteroid use (data not shown).

## Discussion

In this study, we have shown, for the first time, that the relative numbers of NK (CD56^+^CD3^-^) and NKT-like (CD56^+^CD3^+^) cells in the peripheral blood of COPD subjects are reduced compared to smokers. In addition, and corrected for cell numbers, cytotoxic activity of both NK (CD56^+^CD3^-^) and NKT-like (CD56^+^CD3^+^) cells is significantly reduced and correlates positively with degree of airway obstruction as measured by FEV_1_.

In studying the total number of cells in the peripheral blood, we confirmed the findings of others that there are no significant differences in the overall proportion of CD8^+ ^T lymphocytes between HNS, smokers and COPD subjects [14,35]. However, the proportion of highly cytotoxic T_EMRA _cells, as determined by their high perforin content [18], was lower. The decreased proportion of T_EMRA _cells in COPD subjects and smokers has not been previously reported and appears to be related to smoking *per se*, rather than disease state, although this would need to be confirmed by looking at ex-smokers. One possible explanation for our finding is that these cells could be reduced in the periphery as a result of them trafficking to the lung. Since these changes occurred in both smokers with and without COPD they cannot in themselves be responsible for the disease, but could facilitate the development of disease in smokers in synergy with other inflammatory changes.

The proportion of NK (CD56^+^CD3^-^) cells was significantly reduced in COPD subjects compared to HNS and was reduced, although not significantly, compared to smokers. This reduction in NK cells in COPD subjects has been previously reported [19]. Human NK cells can be sub-divided into two distinct subsets; CD56^dim^CD16^+ ^and CD56^bright^CD16^-^. In the periphery, the majority (~90%) are CD56^dim^CD16^+ ^whereas at sites of inflammation CD56^bright^CD16^-^NK cells predominate. Further analysis of the NK subsets showed a statistically significant proportional increase of CD56^bright^CD16^- ^NK cells, which has not been previously reported in COPD. The CD56^bright^CD16^- ^subset has a lower cytotoxic potential, but has the capacity to secrete cytokines and are therefore regarded as immunoregulatory [36]. The relative increase in this cell subset could signify that NK cells play a role in the pathophysiology of COPD not previously identified. However, without sampling the lung we can only hypothesis as to their role in the disease. No difference in NK cell numbers or functional activity has been found in lung parenchyma of COPD patients [21], although a decrease has been seen in the bronchoalveolar lavage (BAL) of chronic bronchitis patients [13] suggesting that there could be intra-compartmental variability.

The overall proportion of NKT-like (CD56^+^CD3^+^) cells was decreased in COPD subjects. The proportion of these cells that expressed CD8 was increased showing that, similar to NK cells, the subset bias is different in COPD patients, indicating potential selective enrichment or active recruitment to the lung. The immune regulatory role of CD56^+^CD3^+ ^cells remains poorly defined; both for the overall CD56^+^CD3^+ ^cell population and for the phenotypically different CD56^+^CD3^+ ^cell subtypes [37]. Due to the extremely low number of invariant NKT cells in the peripheral blood [25] our analysis was expanded to include both invariant NKT cells and the TCR diverse non-invariant NKT cells by using CD3 and CD56 as markers. These markers, when used in conjunction with CD4 and CD8, enabled the analysis of CD4^+^, CD8^+ ^and double negative (DN) NKT cells. This analysis revealed that in COPD subjects the overall proportion of NKT cells was decreased and the relative proportion of CD8^+ ^NKT cells was increased. Recent studies have highlighted the distinct Th1- and Th2-type cytokine profiles of NKT cell subpopulations [38-42]. The CD4^+ ^NKT cells produce both Th1- and Th2-type cytokines [38,40-42] and the CD8^+ ^and DN NKT cells produce predominantly Th1-type cytokines [39-42], which could influence the cytokine milieu at the site of inflammation, especially as they are prolific producers. No difference in cytotoxic ability of the three NKT-like subsets has been reported to date.

The differential expression of both perforin and granzyme B within the same cell in CD8+ T lymphocytes, CD56^dim^CD16^+ ^NK cells and NKT-like (CD56^+^CD3^+^) cells in COPD subjects has not been previously reported. By measuring the proteins at the same time and in the same cell it was possible to identify that the cells that express both perforin and granzyme B, were reduced in COPD subjects. These are of greatest interest as they would be the most cytotoxic. It is worth mentioning pre-stored perforin and granzyme B are normally only found in T_EMRA _and effector memory cells, but not in naïve or central memory cells [18]. A previous study by Morisette *et al *showed no difference in the levels of perforin or granzyme B in peripheral CD8+ T lymphocytes and CD56+ cells from emphysema patients compared to smokers and healthy controls [43]. Our study goes one stage further and looks at the proportion of cells that express both cytotoxic proteins and our conclusions complement theirs as we too propose that the cytotoxic cells are selectively recruited to the lung, or the cells are activated within the lung, by a hitherto unknown antigen. This hypothesis is also supported by the findings of Hodge *et al *who reported an increase in the percentage of cells expressing either granzyme B or perforin in the airways and periphery of COPD patients [31]. Again, however, only one protein was measured in each cell so the difference that we report would not have been measurable. Hodge also reported an increase in exogenous granzyme B in the lung highlighting another potential role in the pathogenesis of disease. Furthermore, a previous study by Chrysofakis *et al *has shown that the CD8^+ ^T cells contained within the induced sputum of smokers with COPD were more cytotoxic and expressed more perforin than those in smokers and HNS [28]. The characteristic lung tissue destruction witnessed in COPD subjects [27] could, therefore, be partially mediated by the cytotoxic cells identified in this study, as they all express characteristic sets of chemokine receptors and adhesion molecules that are required for homing to inflamed tissues, such as CXCR3, whose ligand IP-10 is known to be up-regulated in COPD lung epithium [44].

We have also demonstrated that there is a significant decrease in the cytotoxic activity of peripheral blood NK (CD56^+^CD3^-^) and NKT-like (CD56^+^CD3^+^) cells in COPD subjects, compared to smokers and HNS. The measured reduction in cytotoxic activity is not related to absolute cell numbers, as this is accounted for in the assay, but appears to be related to the numbers of NK (CD56^+^CD3^-^) and NKT-like (CD56^+^CD3^+^) cells that expressed both perforin and granzyme B. In the 4 hour LDH release assay performed, the majority of killing measured would be a result of these double positive cells. The perforin only cells, whilst theoretically capable of killing *in vivo*, due to their ability to form pores, need the synergistic activity of the apoptosis inducing granzyme B to kill within the 4-hour window of the assay. Granzyme B only cells could kill through the endocytotic uptake of granzyme B by the target cell, but this would be less effective than the perforin and granzyme B combination.

The dose dependant reduction of cytotoxic activity of the NK (CD56^+^CD3^-^) and NKT-like (CD56^+^CD3^+^) cells when incubated with either an anti-perforin antibody alone or in combination with an anti-granzyme B antibody confirmed that the measured killing was a result of the granule exocytosis pathway.

We believe that the measurement of peripheral blood NK and NKT-like cell activity could be a potential biomarker as it shows a significant difference between smokers with and without disease and correlates with FEV_1_. We acknowledge that it does not allow us to draw any conclusions about the mechanism of disease since we have only studied peripheral cells. The present study has some limitations that deserve comment. Firstly, six patients received inhaled corticosteroids. For this reason, the results obtained in patients receiving or not receiving inhaled corticosteroids were compared, and no significant differences were found. Secondly, although the range of smoking histories overlapped (between smokers and COPD subjects) there was no overlap in terms of the data for cytotoxicity, perforin and granzyme B expression and cell numbers, showing that the differences were likely to be independent of smoking *per se *and related to disease. The caveat of relatively low participant numbers should also be mentioned, however, the high significance and tight groupings of data, belay at least some of that caution. Regression analysis confirmed that all the key differences reported here, between the COPD subjects, smokers and HNS, remained, even after adjusting for age and gender (data not shown).

## Conclusion

In summary, these experiments have shown that there are significant differences in the proportions, subsets, intracellular proteins and cytotoxic abilities of CD56^+^CD3^- ^(natural killer; NK) cells and CD56^+^CD3^+ ^(NKT-like) cells in the peripheral blood of COPD subjects.

## Competing interests

The authors declare that they have no competing interests.

## Authors' contributions

RAU carried out the experimental work and wrote the manuscript. JRL and IT participated in the study's design and edited the manuscript. LF and JC conceived the study, participated in its' design and co-ordination, and edited the manuscript. All authors read and approved the final manuscript.
